# The Pro-Oncogenic Sphingolipid-Metabolizing Enzyme β-Galactosylceramidase Modulates the Proteomic Landscape in BRAF(V600E)-Mutated Human Melanoma Cells

**DOI:** 10.3390/ijms241310555

**Published:** 2023-06-23

**Authors:** Davide Capoferri, Paola Chiodelli, Marzia Corli, Mirella Belleri, Elisa Scalvini, Luca Mignani, Jessica Guerra, Elisabetta Grillo, Veronica De Giorgis, Marcello Manfredi, Marco Presta

**Affiliations:** 1 Unit of Experimental Oncology and Immunology, Department of Molecular and Translational Medicine, University of Brescia, 25123 Brescia, Italy; davide.capoferri@unibs.it (D.C.); luca.mignani@unibs.it (L.M.);; 2 Department of Translational Medicine, University of Piemonte Orientale, 28100 Novara, Italymarcello.manfredi@uniupo.it (M.M.); 3 Center for Allergic and Autoimmune Diseases, University of Piemonte Orientale, 28100 Novara, Italy; 4 Consorzio Interuniversitario Biotecnologie (CIB), Unit of Brescia, 25123 Brescia, Italy

**Keywords:** melanoma, proteomics, β-galactosylceramidase

## Abstract

β-Galactosylceramidase (GALC) is a lysosomal enzyme involved in sphingolipid metabolism by removing β-galactosyl moieties from β-galactosylceramide and β-galactosylsphingosine. Previous observations have shown that GALC may exert pro-oncogenic functions in melanoma and *Galc* silencing, leading to decreased oncogenic activity in murine B16 melanoma cells. The tumor-driving BRAF(V600E) mutation is present in approximately 50% of human melanomas and represents a major therapeutic target. However, such mutation is missing in melanoma B16 cells. Thus, to assess the impact of GALC in human melanoma in a more relevant *BRAF*-mutated background, we investigated the effect of *GALC* overexpression on the proteomic landscape of A2058 and A375 human melanoma cells harboring the BRAF(V600E) mutation. The results obtained by liquid chromatography-tandem mass spectrometry (LC-MS/MS) demonstrate that significant differences exist in the protein landscape expressed under identical cell culture conditions by A2058 and A375 human melanoma cells, both harboring the same BRAF(V600E)-activating mutation. GALC overexpression resulted in a stronger impact on the proteomic profile of A375 cells when compared to A2058 cells (261 upregulated and 184 downregulated proteins versus 36 and 14 proteins for the two cell types, respectively). Among them, 25 proteins appeared to be upregulated in both A2058-upGALC and A375-upGALC cells, whereas two proteins were significantly downregulated in both GALC-overexpressing cell types. These proteins appear to be involved in melanoma biology, tumor invasion and metastatic dissemination, tumor immune escape, mitochondrial antioxidant activity, endoplasmic reticulum stress responses, autophagy, and/or apoptosis. Notably, analysis of the expression of the corresponding genes in human skin cutaneous melanoma samples (TCGA, Firehose Legacy) using the cBioPortal for Cancer Genomics platform demonstrated a positive correlation between GALC expression and the expression levels of 14 out of the 27 genes investigated, thus supporting the proteomic findings. Overall, these data indicate for the first time that the expression of the lysosomal sphingolipid-metabolizing enzyme GALC may exert a pro-oncogenic impact on the proteomic landscape in *BRAF*-mutated human melanoma.

## 1. Introduction

β-Galactosylceramidase (GALC; EC 3.2.1.46) is a lysosomal acid hydrolase that catalyzes the removal of the β-galactose moiety from β-galactosylceramide and other sphingolipids [1]. Recent observations have shown that a progressive increase in GALC expression occurs during melanoma progression in human pathological skin specimens ranging from common nevi to stage IV melanoma [1]. These data suggest that GALC might act as an oncogenic enzyme during melanoma progression. In keeping with this hypothesis, *Galc* knockdown causes a decrease in the tumorigenic and metastatic potential of murine melanoma B16 cells that also showed significant alterations in their lipidomic profile, characterized by increased levels of the oncosuppressive sphingolipid ceramide and of diacylglycerols, mirrored by a decrease in sphingomyelins, phosphatidylethanolamines, and cholesteryl esters. Accordingly, increased levels of ceramide were observed in GALC-silenced human melanoma A2058 cells and tumor xenografts, with a consequent decrease in their tumorigenic potential [1]. However, the mechanism(s) by which GALC exerts its pro-tumorigenic functions in melanoma remains poorly understood.

Mass spectrometry (MS)-based proteomics has been emerging as a core technique for largescale protein characterization in cells and tissue samples by providing a qualitative and quantitative analysis of proteins produced under different physiological and pathological conditions, including cancer [2]. Recently, analysis of the proteome has been considered as a tool for the advancement of diagnostic and prognostic biomarkers in melanoma, as well as for the identification of biological pathways leading to melanoma progression [3,4,5].

The BRAF(V600E)-activating mutation is present in approximately 50% of human melanomas and represents a major target for melanoma therapy [6]. However, such mutation is missing in murine melanoma B16 cells [7]. To obtain further insights into the role of GALC in human melanoma, liquid chromatography-tandem mass spectrometry (LC-MS/MS) was used in the present work to investigate the impact of *GALC* overexpression on the proteomic profile of BRAF-mutated human melanoma cells. To this aim, *GALC* was stably overexpressed in BRAF(V600E)-mutated A2058 and A375 human melanoma cells that express intermediate levels of *GALC* when compared to other human melanoma cell lines (Supplementary Figure S1). The use of two cell lines harboring the same driver mutation appeared to be necessary given the well-known tumor heterogeneity and would have allowed us to define common and individual protein profiles modulated by *GALC* overexpression in BRAF-mutated human melanoma cells.

The results of the present work extend previous observations about a pro-oncogenic role of GALC in *Braf* wildtype murine melanoma cells [1] by demonstrating that *GALC* overexpression increases the tumorigenic potential of human melanoma cells harboring the tumor-driving BRAF(V600E) mutation. Moreover, LC-MS/MS proteomic analysis, supported by transcriptomic data mining, demonstrates for the first time that *GALC* upregulation exerts a significant impact on the proteomic landscape of *BRAF*-mutated human melanoma cells, leading to the modulation of the expression of proteins involved in different aspects of tumor progression, including endoplasmic reticulum responses, the metastatic process, and tumor immune escape.

## 2. Results

### 2.1. GALC Overexpression in A2058 and A375 Melanoma Cells

**Figure 1:**
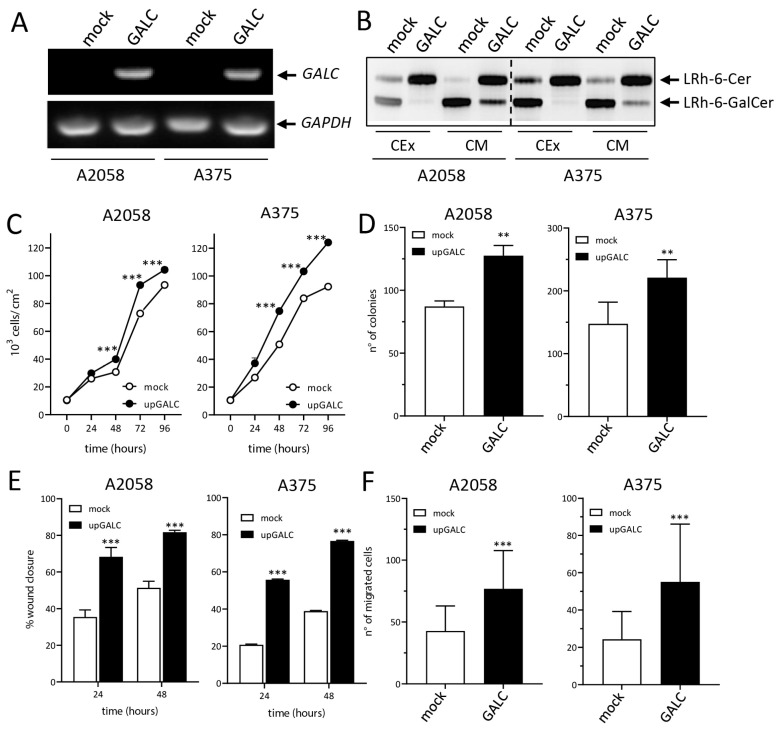
GALC upregulation affects the proliferative and migratory potential of human melanoma cells. RT-PCR (**A**) and enzymatic activity TLC (**B**) assays show the increased expression and activity of GALC in A2058-upGALC and A375-upGALC cell extracts (CEx) and conditioned media (CM) when compared to mock cells. GALC upregulation stimulates the growth (**C**) and colony formation capacity (**D**) of A2058 and A375 cells. GALC upregulation stimulates the migratory potential of A2058 and A375 cells after a mechanical scratch of the cell monolayer (**E**) and in a Boyden chamber chemotaxis assay (**F**). Data are the mean ± SEM, ** *p* < 0.01, *** *p* < 0.001.

A2058-upGALC and A375-upGALC cells, together with the corresponding control A2058-mock and A375-mock cells, were obtained by lentiviral infection, and *GALC* overexpression was confirmed by semiquantitative RT-PCR and enzymatic activity assays (Figure 1A,B). As shown in Figure 1C,D, A2058-upGALC and A375-upGALC cells showed a significant increase in their proliferative potential and their anchorage-independent growth ability when compared to the corresponding mock cells. In addition, *GALC*-overexpressing cells were characterized by increased motility when assessed in wound healing and Boyden chamber assays (Figure 1E,F). Together, these data indicate that *GALC* upregulation exerts a pro-oncogenic function on both A2058 and A375 cells. On this basis, LC-MS/MS proteomic analysis was performed on the cell extracts of mock and upGALC cells originating from both cell lines.

### 2.2. Analysis of A2058 and A375 Cell Proteomics

**Figure 2:**
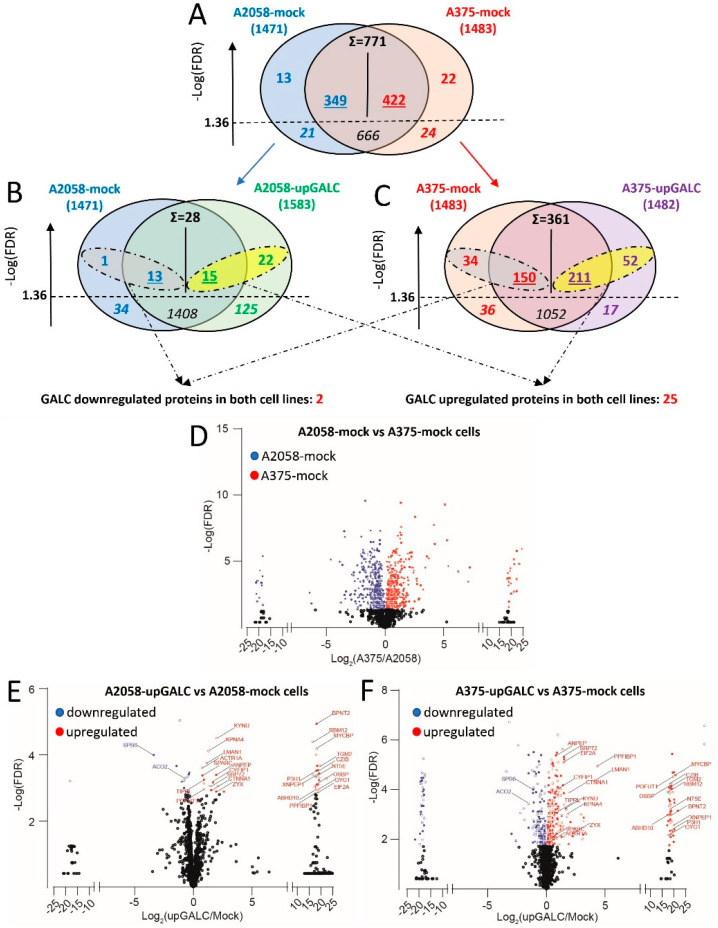
Summary of the quantitative data obtained by proteomic analysis of mock and upGALC cells. (**A**–**C**) Comparison of protein counts identified for (**A**) A2058-mock vs. A375-mock cells, (**B**) A2058-mock vs. A2058-upGALC cells, and (**C**) A375-mock vs. A375-upGALC cells. Each Venn diagram shows the breakdown of protein counts between the two groups. Σ: total number of proteins differentially expressed in the two groups. Underlined numbers: proteins expressed at significantly higher levels in the corresponding group. Numbers in italics: proteins expressed at the same level in the two groups. Numbers in bold above the −Log(FDR) threshold value of 1.36 represent the number of proteins detected at a significant level in only one group, whereas those below the threshold value (bold and italics) represent the number of proteins detected in only one group but below the confidence level. (**D**,**E**) Volcano plot representation of proteins differentially expressed in A2058-mock vs. A375-mock cells (**D**), A2058-upGALC vs. A2058-mock cells (**E**), A375-upGALC vs. A375-mock cells (**F**).

Given the well-known heterogeneity of the proteomic landscape even among cell lines originating from the same tumor type [8], a preliminary analysis was performed to compare the proteomic profile of A2058-mock and A375-mock cells. LC-MS/MS resulted in the identification (protein-level FDR below 1%) of 1471 and 1483 proteins for mock A2058 and A375 cells, respectively (Figure 2A,D). Among them, 1437 proteins were detected in both cell types. Comparative quantitative analysis of averaged spectral count values for the identified proteins resulted in 666 proteins equally expressed and 771 proteins differentially expressed in the two cell types. Among the differentially expressed proteins (Supplementary Table S1), 349 proteins were expressed at higher levels in A2058-mock cells and 422 proteins in A375-mock cells. Proteins that showed expression levels above the sensitivity threshold of the LC-MS/MS procedure in only one of the two cell types (13 and 22 proteins for A2058-mock and A735-mock cells, respectively) were included in the corresponding list of upregulated proteins, resulting in 362 and 444 entries for A2058-mock cells and A375-mock cells, respectively.

**Figure 3:**
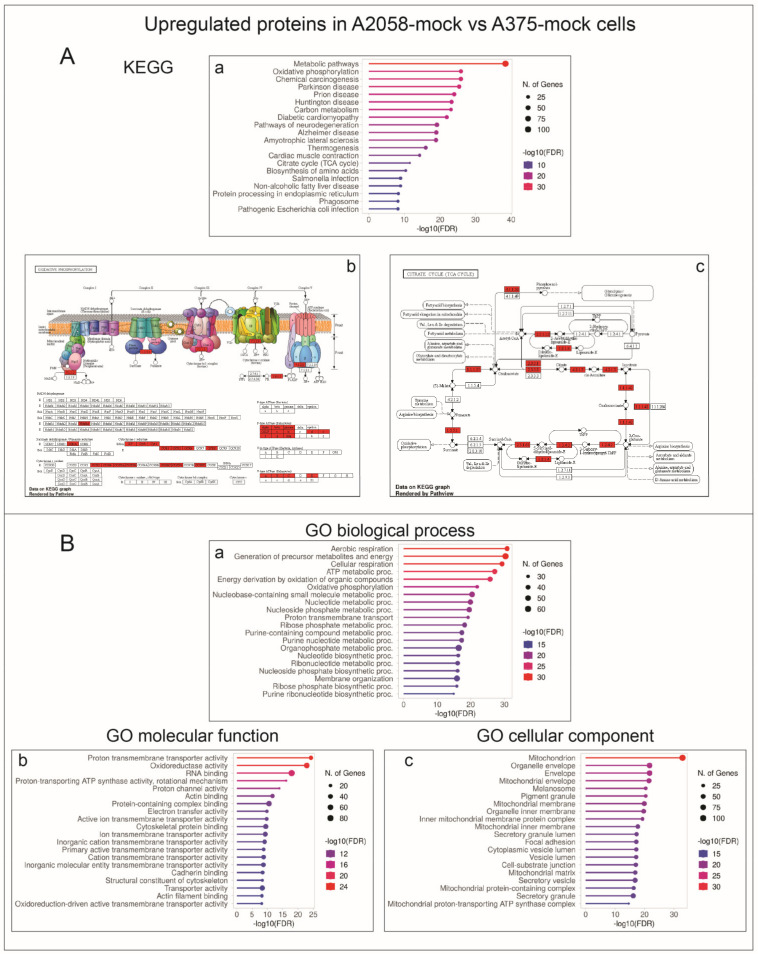
KEGG and Gene Ontology annotation of proteins expressed at higher levels in A2058-mock vs. A375-mock cell extracts. (**A**) Enriched KEGG pathways are related to energetic metabolism, including oxidative phosphorylation, mitochondrial function, and the TCA cycle (a). Pathview rendering of oxidative phosphorylation (b) and TCA cycle (c) KEGG pathways showing the proteins expressed at higher levels in A2058-mock vs. A375-mock cell extracts (in red). (**B**) Significantly enriched GO biological process (a), molecular function (b), and cellular component (c) terms are related to energetic processes associated with mitochondrial activity.

When analyzed with the gene-set enrichment tool ShinyGO [9], A2058-mock cells showed higher levels of expression for proteins associated with KEGG pathways related to energetic metabolism when compared to A375-mock cells, including, among others, oxidative phosphorylation, mitochondrial function, and TCA cycle (Figure 3A). Accordingly, the Gene Ontology (GO) molecular function, biological process, and cellular component terms also related to energetic processes associated with mitochondrial activity were significantly enriched in the set of the 362 proteins more expressed in A2058-mock cells (Figure 3B).

**Figure 4:**
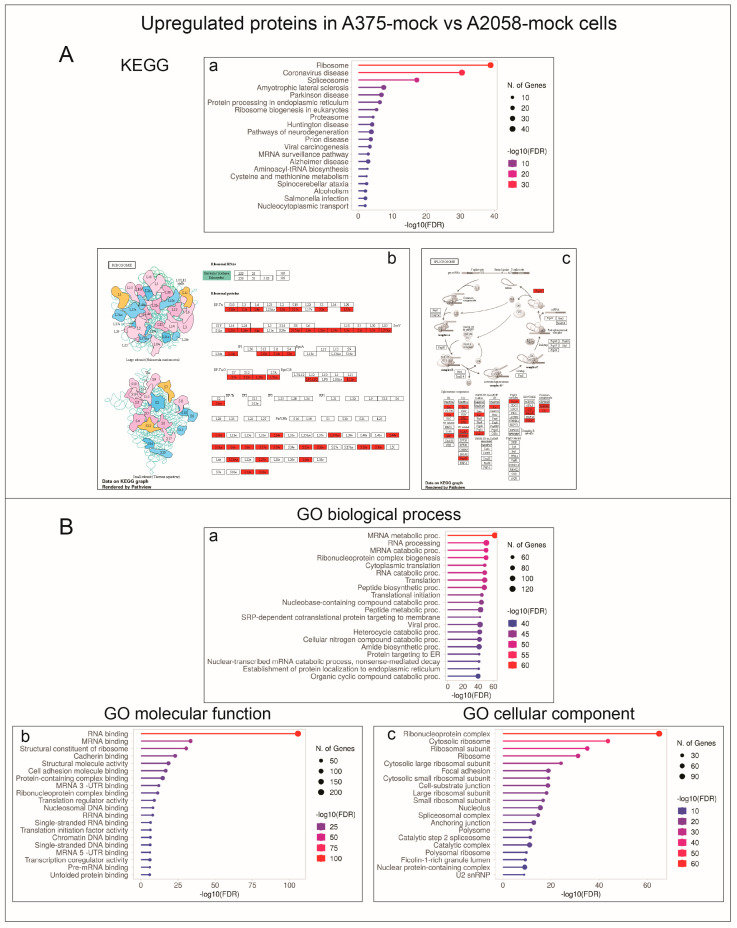
KEGG and Gene Ontology annotation of proteins expressed at higher levels in A375-mock vs. A2058-mock cell extracts. (**A**) The proteins expressed at higher levels in A375-mock cells are significantly associated with the ribosome and spliceosome KEGG pathways (a). Pathview rendering of the ribosome (b) and spliceosome (c) KEGG pathways showing the proteins expressed at higher levels in A375-mock vs. A2058-mock cell extracts (in red). (**B**) Significantly enriched GO biological process (a), molecular function (b), and cellular component (c) terms refer to categorizations related to mRNA binding/splicing and ribosomes.

At variance, the 444 proteins expressed at higher levels in A375-mock cells were more significantly associated with the ribosome and spliceosome KEGG pathways (Figure 4A). In keeping with these findings, the corresponding enriched GO terms referred to categorizations related to mRNA binding/splicing and ribosomes (Figure 4B).

**Figure 5:**
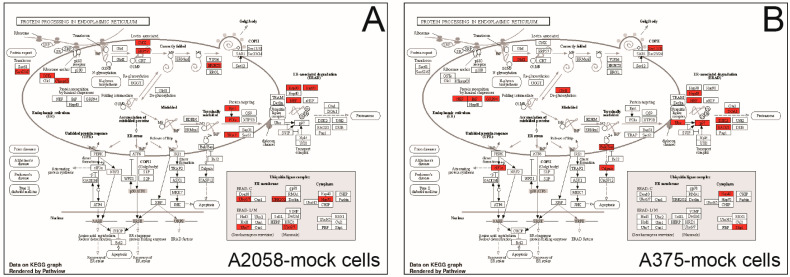
A2058-mock and A375-mock cells differentially express proteins involved in the protein processing that occurs in the ER. Pathview rendering of the protein processing in endoplasmic reticulum KEGG pathway showing the proteins expressed at higher levels in A2058-mock (**A**) and A375-mock (**B**) cell extracts (in red).

Of note, A2058-mock and A375-mock cells differentially express proteins involved in the protein processing that occurs in the endoplasmic reticulum (ER). Indeed, A2058-mock cells express higher levels of proteins belonging to the ubiquitin ligase complex, whereas A375-mock cells express higher levels of proteins related to ER-associated degradation (Figure 5).

Together, these data indicate that significant differences exist in the protein landscape expressed under identical cell culture conditions by A2058 and A375 human melanoma cells, both harboring the same BRAF(V600E)-activating mutation.

### 2.3. Impact of GALC Overexpression on the Proteomic Profile of A2058 and A375 Cells

As observed for mock cells, LC-MS/MS analysis identified 1583 and 1482 proteins in A2058-upGALC and A375-upGALC cell extracts, respectively (Figure 2B,C). When compared to the corresponding control A2058-mock cells, 37 proteins were upregulated, and 14 proteins were downregulated upon *GALC* transduction in A2058 cells, whereas the levels of expression of 1408 proteins remained unchanged (Figure 2B,E and Supplementary Table S2). At variance, *GALC* overexpression in A375 cells resulted in the upregulation of the levels of 263 proteins and in the downregulation of 184 proteins, while 1052 proteins remained unchanged (Figure 2C,F and Supplementary Table S3). Thus, *GALC* overexpression resulted in a stronger impact on the proteomic profile of A375 cells when compared to A2058 cells (*p* < 0.0001, chi-square test).

**Figure 6:**
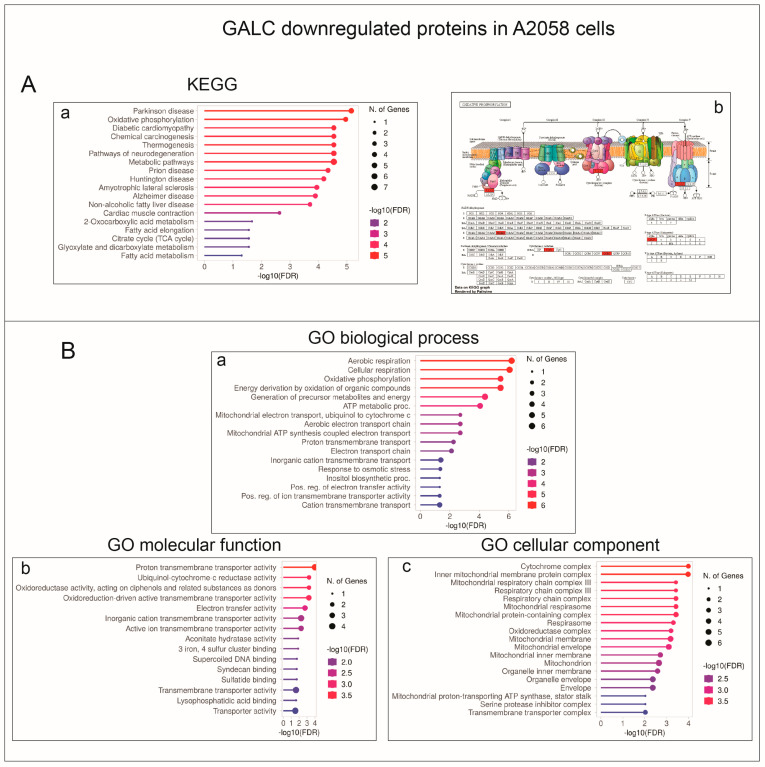
KEGG and Gene Ontology annotation of proteins downregulated following GALC overexpression in A2058-upGALC cells. (**A**) Enriched KEGG pathways are related to energetic metabolism, including oxidative phosphorylation (a). Pathview rendering of the oxidative phosphorylation KEGG pathways (b) showing the proteins expressed at higher levels in A2058-upGALC vs. mock cell extracts (in red). (**B**) Significantly enriched GO biological process (a), molecular function (b), and cellular component (c) terms are related to mitochondrial processes, including oxidative phosphorylation, mitochondrial respiratory chain complexes, and aerobic respiration.

ShinyGO categorization analysis of the 37 proteins upregulated in A2058-upGALC cells did not allow unambiguous identification of enriched GO terms, with just three entries associated with the “nicotinate and nicotinamide metabolism” KEGG. At variance, despite their limited number, the 14 downregulated proteins were found to belong to enriched KEGG pathways and GO terms related to mitochondrial processes, including oxidative phosphorylation, mitochondrial respiratory chain complexes, and aerobic respiration (Figure 6).

**Figure 7:**
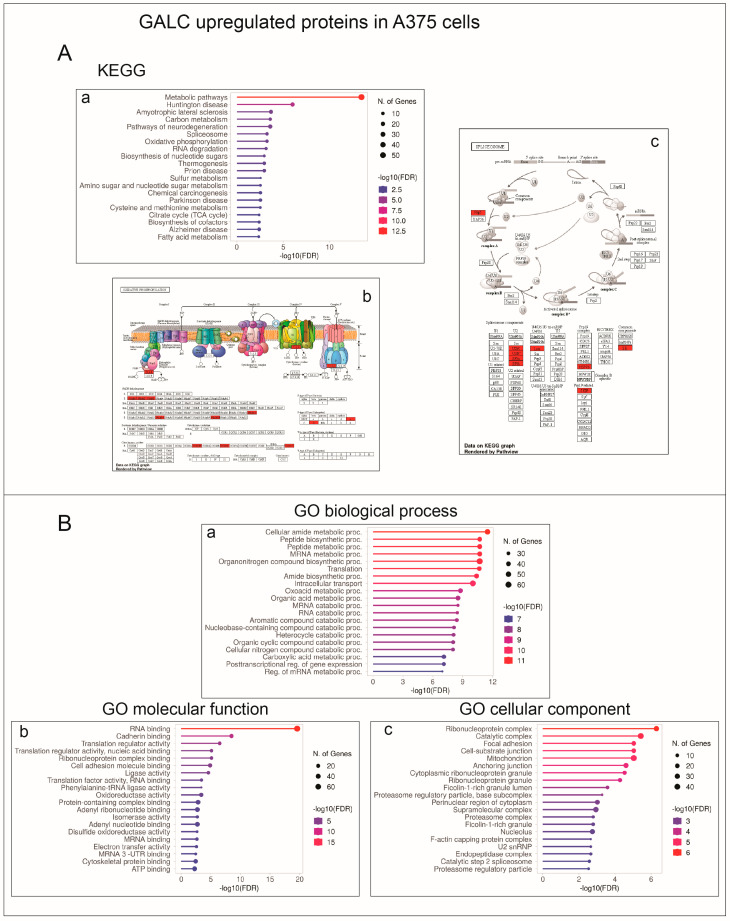
KEGG and Gene Ontology annotation of proteins upregulated following GALC overexpression in A375-upGALC cells. (**A**) Enriched KEGG pathways are related to metabolic pathways, including oxidative phosphorylation and spliceosome (a). Pathview rendering of oxidative phosphorylation (b), and spliceosome (c) KEGG pathways showing the proteins expressed at higher levels in A375-upGALC vs. mock cell extracts (in red). (**B**) Significantly enriched GO biological process (a), molecular function (b), and cellular component (c) terms are related to the RNA metabolism/ribonucleoprotein complex and various metabolic processes.

Concerning A375-upGALC cells, categorization analysis indicated that upregulated proteins were mainly associated with spliceosome and oxidative phosphorylation KEGG pathways and to GO terms related to the RNA metabolism/ribonucleoprotein complex and to various metabolic processes (Figure 7).

**Figure 8:**
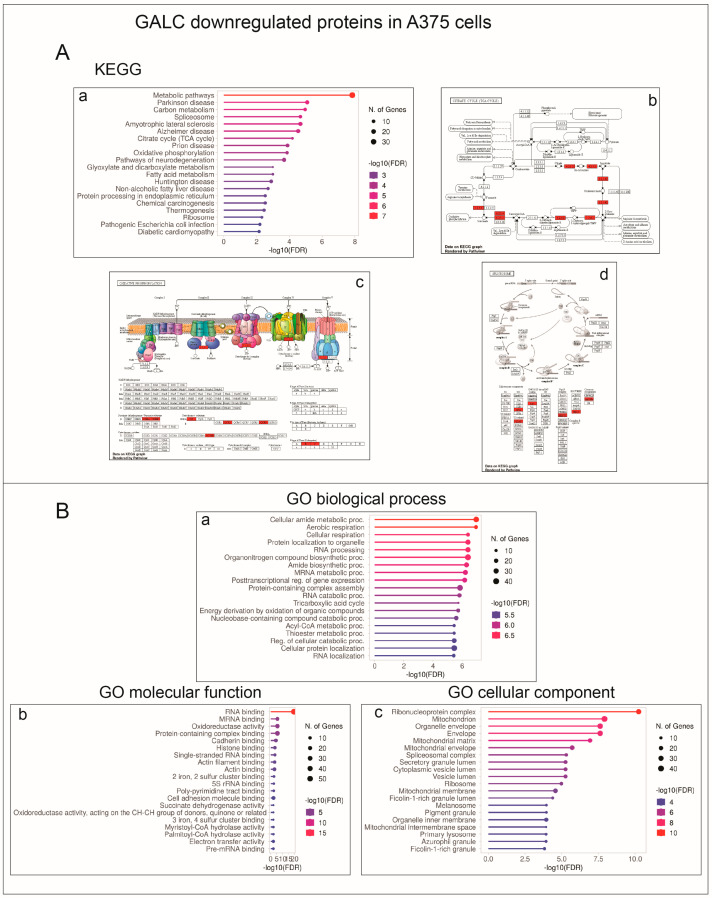
KEGG and Gene Ontology annotation of proteins downregulated following GALC overexpression in A375-upGALC cells. (**A**) Enriched KEGG pathways are related to metabolic pathways, including oxidative phosphorylation and spliceosome (a). Pathview rendering of TCA cycle (b), oxidative phosphorylation (c) and spliceosome (d) KEGG pathways showing the proteins expressed at lower levels in A375-upGALC vs. mock cell extracts (in red). (**B**) Significantly enriched GO biological process (a), molecular function (b), and cellular component (c) terms refer mainly to mRNA binding/splicing as well as aerobic respiration and mitochondrion.

Notably, the 184 proteins downregulated in A375-upGALC cells were also more significantly associated with the spliceosome and TCA cycle/oxidative phosphorylation KEGG pathways. Accordingly, enriched GO terms belonging to biological process, molecular function, and cellular component categorizations of these downregulated proteins referred mainly to mRNA binding/splicing as well as aerobic respiration and mitochondrion (Figure 8).

Overall, this categorization analysis suggests that *GALC* upregulation modulates the protein landscape in melanoma cells by affecting the biological processes related to RNA metabolism and mitochondria function.

Based on these premises, we investigated which proteins were downregulated or upregulated by GALC transduction in both cell lines by comparing the entries shown in Supplementary Tables S2 and S3. Twenty-five proteins appeared to be upregulated in both A2058-upGALC and A375-upGALC cells, whereas only two proteins were significantly downregulated in both GALC-overexpressing cell types. The list of these proteins and a brief description of their biological function(s) in cancer (including human melanoma when available) are shown in Table 1.

Notably, 6 out of the 27 proteins modulated by *GALC* overexpression in both A2058-upGALC and A375-upGALC cells have been involved in melanoma biology (i.e., aminopeptidase N (CD13), lectin mannose-binding 1,5′-nucleotidase ecto (CD73), secreted protein acidic and cysteine rich, transglutaminase 2, and zyxin, encoded, respectively, by *ANPEP*, *LMAN1*, *NT5E*, *SPARC*, *TGM2*, and *ZYX* genes), 8 proteins have been involved in tumor invasion and metastatic dissemination (i.e., aminopeptidase N (CD13), cytoplasmic FMR1-interacting protein 1, catenin alpha 1, oxysterol-binding protein, prolyl 3-hydroxylase 1, PPFIA-binding protein 1, secreted protein acidic and cysteine rich, and zyxin, encoded, respectively, by *ANPEP*, *CYFIP1*, *CTNNA1*, *OSBP*, *P3H1*, *PPFIBP1*, *SPARC*, and *ZYX* genes), 4 proteins have been implicated in tumor immune escape (i.e., karyopherin subunit alpha 4, kynureninase, 5′-nucleotidase ecto (CD73), and RNA-binding motif protein 12, encoded, respectively, by *KPNA4*, *KYNU*, *NT5E*, and *RBM12* genes), and 10 proteins have been shown to play a role in ER stress responses, mitochondrial antioxidant activity, autophagy, and/or apoptosis (i.e., lectin mannose-binding 1, eukaryotic translation initiation factor 2A, signal recognition particle 72, actin-related protein 1A, abhydrolase domain-containing 10 (depalmitoylase), glycogenin 1, aconitase 2, serpin family B member 6, TOR signaling pathway regulator, and X-prolyl aminopeptidase 1, encoded, respectively, by *LMAN1*, *EIF2A*, *SRP72*, *ACTR1A*, *ABHD10*, *GYG1*, *ACO2*, *SERPINB6*, *TIPRL,* and *XPNPEP1* genes) (see Table 1 and references therein).

Next, RT-qPCR analysis was performed on A2058-upGALC vs. A2058-mock cells to assess the expression levels of genes encoding for various proteins up- or downregulated by *GALC* overexpression in *BRAF*-mutated melanoma cells. As shown in Supplementary Figure S2, the results of RT-qPCR analysis were congruent with proteomic data.

**Figure 9:**
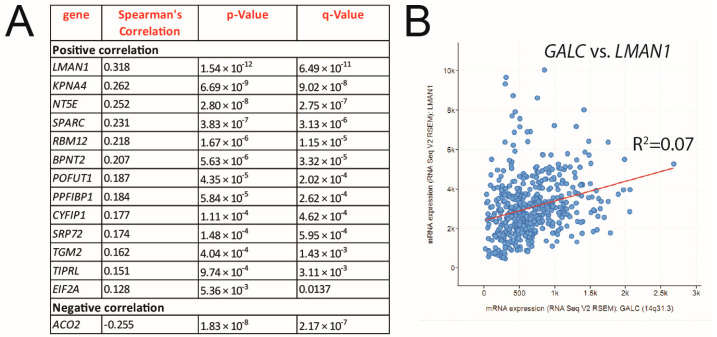
*GALC* expression in human melanoma specimens correlates with the expression of proteins identified by proteomic analysis of upGALC vs. mock melanoma cells. Correlation of *GALC* expression with the expression of the genes encoding for the proteins similarly modulated by *GALC* upregulation in A2058 and A375 cells was assessed in 448 human skin cutaneous melanoma samples (TCGA, Firehose Legacy) using the cBioPortal for Cancer Genomics platform (**A**). Correlation between *GALC* and *LMAN1* expression in human melanoma (**B**).

Finally, the correlation between *GALC* mRNA levels and the expression of the genes encoding for the 27 proteins similarly modulated by *GALC* upregulation in A2058 and A375 cells was assessed in 448 human skin melanoma samples (TCGA, Firehose Legacy) using the cBioPortal for Cancer Genomics platform [38,39]. As shown in Figure 9, the expression levels of 14 out of the 27 genes investigated show a significant correlation with GALC expression in human melanoma specimens, congruent with the proteomic data.

## 3. Discussion

GALC is a lysosomal enzyme involved in sphingolipid metabolism by removing β-galactose from β-galactosylceramide and other terminal β-galactose-containing sphingolipids. Recent observations indicate that this enzyme might be involved in tumor progression (reviewed in [40]). In keeping with this hypothesis, immunohistochemical data have shown that high levels of GALC immunoreactivity are associated with poor prognosis in colorectal cancer patients [41] and that higher GALC expression levels in circulating lung cancer cells correlate with a poor response to therapy, representing a possible predictor biomarker in these patients [42]. In line with these findings and with the observation that sphingolipid metabolic reprogramming plays an important role in melanoma progression [43], analysis of human specimens ranging from common nevi to stage IV melanoma demonstrated a gradual increase in GALC expression during tumor progression that goes along with a decrease in ceramide levels [1]. Accordingly, *Galc* silencing results in significant inhibition of the tumorigenic and metastatic activity of *Braf* wildtype murine melanoma B16-F10 cells that showed alterations in their sphingolipid profile, characterized by an increase in the intracellular levels of the oncosuppressor sphingolipid ceramide. A similar ceramide accumulation was observed in human melanoma cells following GALC downregulation [1].

Here, we extend these observations and demonstrate that GALC overexpression plays a pro-tumorigenic function on both A2058 and A375 human melanoma cells that harbor the BRAF(V600E)-activating mutation, which is present in approximately 50% of human melanomas [6]. A2058 and A375 cells express intermediate levels of *GALC* mRNA and protein when compared to other human melanoma cell lines (Supplementary Figure S1), being therefore suitable for assessing the impact of the upregulation of this enzyme on the biological behavior of human melanoma cells in a BRAF-mutated background. Indeed, our data indicate that *GALC* upregulation induces a significant increase in the proliferative potential and anchorage-independent growth of both *BRAF*-mutated human cell lines, paralleled by increased cell motility in a Boyden chamber assay and after in vitro wounding of the cell monolayer.

These findings prompted us to investigate the impact of *GALC* upregulation on the proteomic landscape of both A2058 and A375 human melanoma cells. The results of the LC-MS/MS analysis of the cell extracts of control and *GALC*-overexpressing cells indicate that significant differences exist in the protein landscape expressed under identical cell culture conditions by the two melanoma cell lines that harbor the same driver mutation. Indeed, in keeping with the well-known heterogeneity of the proteomic landscape, even among cell lines originating from the same tumor type [8], 771 proteins (52%) out of 1437 proteins detected in both control A2058 and A375 cells were present at different levels in the two cell types. Notably, KEGG and GO categorizations indicated that A2058-mock cells express higher levels of proteins related to energy metabolism and mitochondrial activity, whereas proteins related to mRNA binding/splicing and ribosome terms are present at higher levels in A375-mock cell extracts. Experimental evidence indicates that BRAF-driven ER stress and unfolded protein response play an important role in melanoma (reviewed in [44]). In this frame, a further indication of melanoma cell heterogeneity derives from the observation that A2058-mock and A375-mock cells differentially express proteins involved in the protein processing that occurs in the ER, A2058-mock cells expressing higher levels of proteins belonging to the ubiquitin ligase complex, whereas A375-mock cells are characterized by higher levels of proteins related to the ER-associated protein degradation process.

Based on this cell heterogeneity, it is not surprising that GALC overexpression exerted a different impact on the proteomic landscape of the two melanoma cell lines. Indeed, *GALC* transduction in A2058 cells resulted in the up- or downregulation of the expression levels of 37 and 14 proteins, respectively, whereas it exerted a stronger impact on A375 cells (263 and 184 proteins up- or downregulated, respectively). At present, the mechanisms responsible for such differences remain unknown. It will be interesting to evaluate the impact exerted by GALC overexpression on the sphingolipidomic profile of the two cell lines.

Despite the differences observed between the two cell lines in terms of the number of proteins whose levels are modulated by *GALC* upregulation, categorization analysis indicates a significant enrichment in both cell lines of downmodulated proteins involved in mitochondrial functions, including oxidative phosphorylation, mitochondrial respiratory chain complexes, and aerobic respiration. Melanoma cells can shuttle between glycolysis and respiration depending upon conditions of growth, hypoxia, acidosis, and therapy, and BRAF activity has been shown to suppress oxidative phosphorylation, thus driving aerobic glycolysis in melanoma (see [45] and references therein). Thus, GALC appears to modulate the energetic plasticity of melanoma cells by metabolic reprogramming. In this frame, it is interesting to note that alterations in the sphingolipid metabolism via modulation of the expression levels of the lysosomal acid ceramidase affect mitochondria activity in melanoma cells [46]. Further studies will be required to elucidate the impact of GALC on the rewiring of energetic metabolism in melanoma.

Among the proteins whose levels of expression were affected by *GALC* overexpression in melanoma cells, 25 of them were upregulated in both A2058-upGALC and A375-upGALC cells, whereas only 2 of them were downregulated in both cell types. Of note, six proteins are known to play a significant role in human melanoma. Indeed, aminopeptidase N (CD13) promotes melanoma growth, angiogenesis, and metastatic dissemination [13,14]. Similarly, the cysteine-rich acidic matrix-associated protein (SPARC) plays a role in melanoma metastasis [29], whereas lectin mannose-binding 1 (LMAN1) is involved in melanoma ER stress and autophagy [21], and the expression of the zinc-binding, focal adhesion-associated phosphoprotein zyxin has been shown to affect melanoma cell spreading and proliferation [34]. Finally, transglutaminase 2 plays a role in melanoma radioresistance [31], whereas the plasma membrane protein 5′-nucleotidase ecto (CD73) may favor melanoma immune escape via the CD73/adenosine axis [23].

Together with CD13, SPARC, and zyxin, GALC-upregulated cytoplasmic FMR1-interacting protein 1 and catenin alpha 1 are also implicated in the metastatic process by regulating cytoskeletal dynamics and cell adhesion of tumor cells [15,16], PPFIA-binding protein 1 drives tumor cell migration and invasion via the FAK/Src/JNK pathway [27], ER-associated collagen prolyl 3-hydroxylase 1 plays a pivotal role in cancer cell proliferation, migration, and invasion [25], and the lipid transporter oxysterol-binding protein has been proposed as a potential marker for cholangiocarcinoma metastasis [24]. Together, these data suggest that GALC may modulate the metastatic potential of melanoma cells. In keeping with this hypothesis, *GALC* is expressed at higher levels in human melanoma metastases when compared to primary tumors, and *Galc* knockdown hampers the capacity of murine melanoma B16-F10 cells to form experimental lung metastases [1].

Immune system evasion represents a hallmark of melanoma progression [47]. Our data indicate that, besides CD73, *GALC* transduction induces an increase in the levels of the importin karyopherin subunit alpha 4, kynureninase, and RNA-binding motif protein 12, all involved in tumor immune escape [19,20,28]. In keeping with a possible role for alterations in the sphingolipid metabolism in immune evasion, sphingolipid pathway enzymes have been shown to modulate immune cell function in cancer [48], sphingomyelin appears to play a key role in tumor progression and immune evasion [49], and neutral sphingomyelinase 2 expression impairs melanoma growth by enhancing CD8+ T-cell responses [50]. Whether and how GALC represents a key player in modulating immune responses in melanoma remains to be investigated.

A possible role of GALC in ER functions in cancer is supported by the observation that GALC overexpression induces not only the upregulation of the levels of the overmentioned LMAN1 that functions as a cargo receptor for glycoprotein transport in the ER [21] but also of the levels of the eukaryotic translation initiation factor 2A involved in ER stress in cancer via the (PERK)-eIF2a-ATF4-CHOP signaling axis [17], the signal recognition particle 72 that mediates the targeting of secretory proteins to the ER [30], and the actin-related protein 1A implicated in the ER-to-Golgi transport and lysosome/endosome movement, representing a possible biomarker for pituitary and colon cancers [11,12].

In keeping with the hypothesis that GALC may modulate the energetic plasticity of melanoma cells (see above), both *GALC*-transduced A2058 and A375 cells are characterized by higher levels of the mitochondrial abhydrolase domain-containing 10 able to affect the mitochondrial antioxidant activity [10] and of glycogenin 1, a glycosyltransferase involved in the first steps of glycogen synthesis downregulated in liver cancers [18]. In addition, *GALC* upregulation causes the downregulation of the oncosuppressor aconitase 2, which affects the TCA cycle and mitochondrial oxidative metabolism in cancer cells [35,36], and of the serine protease inhibitor serpin family B member 6, whose dysregulation is associated with autophagic and apoptotic induction in cancer [37].

Finally, GALC-upregulated proteins include the TOR signaling pathway regulator, an allosteric regulator of the serine/threonine-protein phosphatase 2A in cancer cells [32], and the cytosolic X-prolyl aminopeptidase 1 associated with disease progression and shorter overall survival in multiple myeloma [33], together with 3′(2′), 5′-bisphosphate nucleotidase 2 and CXXC motif-containing zinc-binding protein, whose function(s) in cancer remains unexplored.

In silico analysis of transcriptomic data from 448 human skin melanoma samples performed on the cBioPortal for Cancer Genomics platform [38,39] supported the proteomic data. Indeed, the expression of 14 out of the 27 genes encoding for GALC-modulated proteins in both A2058 and A375 cells was significantly correlated with *GALC* mRNA levels in human melanoma specimens. Among them, three genes encode for proteins related to ER responses (i.e., LMAN1, SRP72, and EIF2A), three genes encode for proteins that play a significant role in the metastatic process (i.e., SPARC, CYFIP1, and PPFIBP1), and three genes encode for proteins involved in tumor immune escape (i.e., KPNA4, NT5E, and RBM12), thus supporting the role of GALC in different aspects of melanoma progression.

Previous observations have shown that GALC may exert a pro-oncogenic role in *Braf* wildtype murine melanoma cells [1]. The results of the present work confirm and extend these findings by demonstrating that *GALC* overexpression increases the tumorigenic potential of both A2058 and A375 human melanoma cells harboring the tumor-driving BRAF(V600E) mutation. In addition, LC-MS/MS proteomic analysis, supported by transcriptomic data mining, indicates for the first time that GALC may exert a pro-oncogenic impact on the proteomic landscape in *BRAF*-mutated human melanoma cells. Previous observations have shown that *GALC* downregulation may exert profound alterations in the lipidome of murine melanoma and exert a significant increase in ceramide levels in A2058 cells [1]. Exogenous administration of ceramide affects the protein profile of different tumor cell types [51,52], and the lack of GALC activity alters the proteome of the central and peripheral nervous system in *Galc*-null Twitcher mice [53]. At present, we do not know whether the effects observed in *BRAF*-mutated human melanoma cells following *GALC* overexpression are due to an excess of enzyme product(s) and/or a reduction in its substrate(s). Further studies will be required to assess the effect of the modulation of GALC activity on the sphingolipidome of human melanoma cells and how this, in turn, may orchestrate their transcriptomic and proteomic profiles.

## 4. Materials and Methods

### 4.1. Cell Cultures and Lentivirus Infection

A2058 and A375 cells were purchased from ATCC, grown in Dulbecco’s modified Eagle medium (DMEM; Thermo Fisher Scientific, Waltham, MA, USA) supplemented with 10% heat-inactivated fetal bovine serum (FBS), 100 U/mL penicillin, and 100 μg/mL streptomycin (Thermo Fisher Scientific) and maintained at 37 °C and 5% CO_2_ in a humidified incubator. For GALC overexpression, cells were infected with a lentivirus (pLenti PGK GFP Puro (w509-5) was a gift from Eric Campeau and Paul Kaufman, Addgene plasmid #19070) harboring the human GALC cDNA (NM_000153.3), thus generating A2058-upGALC and A375-upGALC cells. Cells transduced with an empty vector were used as controls (A2058-mock and A375-mock cells). For the infection protocol, cells were incubated with lentiviral particles for 7 h in a complete medium containing 8.0 μg/mL of polybrene and selected by adding puromycin (1 μg/mL) 24 h later. Next, GALC overexpression was confirmed by semiquantitative RT-PCR. Briefly, cells were processed, and total RNA was extracted using TRIzol Reagent according to the manufacturer’s instructions (Invitrogen, Waltham, MA, USA). Contaminating DNA was digested using DNAse (Promega, Madison, WI, USA), and 2.0 μg of total RNA was retro-transcribed with MMLV reverse transcriptase (Invitrogen) using random hexaprimers in a final 20 μL volume. Then, 1/10th of the reaction was analyzed by semiquantitative RT-PCR using the following primers: GALC, forward: ATCTCTGCATCCATGCTCCT, reverse: CTGATTTAAAATGCGACCCC; GAPDH, forward: ACGGATTTGGTCGTATTGGG, reverse: TGATTTTGGAGGGATCTCGC. The PCR products were then electrophoresed on a 2% agarose gel and visualized by ethidium bromide staining.

### 4.2. GALC Activity Assay

GALC-mediated hydrolysis of the fluorescent GALC substrate LRh-6-GalCer (Nlissamine-rhodaminyl-6-aminohexanoylgalactosyl ceramide) following its incubation with 20 μg of cell extract or 20 μL of their conditioned medium (50×) was quantified by thin-layer chromatography (TLC) [54]. Briefly, 5 nmoles of LRh-6-GalCer in 3:2 chloroform/methanol was concentrated and dissolved in 5 µL of dimethyl sulfoxide (DMSO) and 25 μL of 0.2 M citrate phosphate buffer, pH 4.4. The enzyme source and water were added to a final volume of 100 μL and incubated overnight at 37 °C. The reaction was extracted with 1.9 mL of 3:2 *v*/*v* chloroform/methanol and 0.4 mL of water. The lower phase was collected and evaporated under nitrogen. Samples were spotted on glass-coated silica gel plates and developed in 25:25:25:9:16 volumes of chloroform/ethyl acetate/*n*-propanol/0.25 M KCl/methanol. The fluorescent ceramide spots (LRh-6-Cer) were visualized under an ultraviolet lamp and photographed.

### 4.3. Cell Proliferation Assay

Cells were seeded at 10^4^ cells/cm^2^ in DMEM supplemented with 2.0% FBS. After 24 h (T0), fresh medium was added, and cells were counted 24–96 h thereafter [1]. The data are the mean ± SEM of three experiments in triplicate.

### 4.4. Soft Agar Assay

Cells (5 × 10^4^) were suspended in 2 mL of medium containing 0.3% agar and applied onto 2 mL pre-solidified 0.6% agar in 35 mm culture dishes (3 dishes per cell line). After 15 days of incubation, cell colonies were observed under a phase contrast microscope and counted [55].

### 4.5. Wound Healing Assay

Confluent cells were scraped with a 200 μL tip to obtain a 2 mm thick denuded area. After 24 and 48 h, wounded monolayers were photographed, and the width of the wounds was quantified by computerized analysis of the digitalized images in three independent sites per group [56]. The experiment was performed twice with similar results.

### 4.6. Boyden Chamber Migration Assay

The chemotaxis assay was performed as described with minor modifications [57]. Briefly, cells (5 × 10^4^ cells) were suspended in 50 μL/well of serum-free DMEM and loaded in the upper compartment of a Boyden chamber containing gelatine-coated polyvinylpyrrolidone-free (PVP-free) polycarbonate filters (8 μm pore size, Costar, Cambridge, MA, USA). A total of 30 μL of 10% FBS-containing DMEM was placed in the lower compartment. After 5 h of incubation at 37 °C, cells that had migrated to the lower side of the filter were stained with H&E. Five random fields were counted for each triplicate sample.

### 4.7. Mass Spectrometry

#### 4.7.1. Sample Preparation

Cell samples were lysed with RIPA buffer and denatured with TFE. The samples were subjected to DTT reduction (200 mM), IAM alkylation (200 mM), and complete trypsin protein digestion. The peptide digests were desalted on the Discovery^®^ DSC-18 solid phase extraction 96-well plate (25 mg/well). After the desalting process, samples were vacuum-evaporated and reconstituted in the mobile phase for analysis [58]. All reagents were from Sigma-Aldrich Inc. (St. Louis, MO, USA).

#### 4.7.2. Proteomic Analysis

The digested peptides were analyzed with a UHPLC Vanquish system (Thermo Scientific, Rodano, Italy) coupled with an Orbitrap Q-Exactive Plus (Thermo Scientific). Peptides were separated by a reverse phase column (Accucore™ RP-MS 100 × 2.1 mm, particle size 2.6 µm) at a flow rate of 0.200 mL/min, with water and acetonitrile as mobile phase A and B, respectively, both acidified with 0.1% formic acid. The analysis was performed using the following gradient: 0–5 min from 2% to 5% B; 5–55 min from 5% to 30% B; 55–61 from 30% to 90% B, and hold for one minute. At 62.1 min, the percentage of B was set to the initial condition of the run at 2% and held for about 8 min in order to equilibrate the column for a total run time of 70 min. The mass spectrometry analysis was performed in positive ion mode. The ESI source was used with a voltage of 2.8 kV. The capillary temperature, sheath gas flow, auxiliary gas, and spare gas flow were set at 325 °C, 45 arb, 10 arb, and 2, respectively. S-lens was set at 70 rf. For the acquisition of spectra, a data-dependent (ddMS2) top 10 scan mode was used. Survey full-scan MS spectra (mass range *m*/*z* 381 to 1581) were acquired with resolution R = 70,000 and AGC target 3 × 10^6^. MS/MS fragmentation was performed using high-energy c-trap dissociation (HCD) with resolution R = 35,000 and AGC target 1 × 10^6^. The normalized collision energy (NCE) was set to 30. The injection volume was 3 μL.

The mass spectra analysis was carried out using MaxQuant software (version 1.6.14). MaxQuant parameters were set as follows: trypsin was selected for enzyme specificity; the search parameters were fixed to an initial precursor ion tolerance of 10 ppm and MS/MS tolerance at 20 ppm; as fixed modification, carbamidomethylation was set, whereas oxidation was set as variable modification. The maximum missed cleavages were set to 2. Andromeda search engine searched the spectra in MaxQuant against the Uniprot_CP_Human_2018 sequence database. Label-free quantification was performed, including a match between runs option with the following parameters: protein and peptide false discovery rate was set to 0.01; the quantification was based on the extracted ion chromatograms, with a minimum ratio count of 1; the minimum required peptide length was set to 7 amino acids. Statistical analyses were performed using MaxQuant software (version 1.6.14) and MetaboAnalyst software (version 5.0) (https://www.metaboanalyst.ca/ (accessed on 24 January 2021)) [59].

### 4.8. Analysis of MS Data

Statistics were performed using Microsoft Excel 365 and GraphPad Prism 8. *p*-values were calculated by a two-tailed uncoupled *t*-test of 4 technical replicates per sample. Setting a false discovery rate of 5% by a two-stage linear step-up procedure of Benjamini, Krieger, and Yekutieli [60] allowed us to obtain lists of significantly differentially abundant proteins whose encoding genes were given to ShinyGO for obtaining pathway analyses through KEGG and Gene Ontology databases.

### 4.9. RT-qPCR Analysis

For the analysis of differentially expressed genes, total RNA was extracted from mock and upGALC A2058 cells as described above. RT-qPCR analysis on retro-transcribed RNA was performed using specific primers (Supplementary Table S4).

## Figures and Tables

**Table 1:** List of common significantly up- and downregulated proteins upon GALC transduction in both A2058 and A375 cell lines. Each entry is completed by the name of the gene encoding for the listed protein and its known referenced biological function.

Protein	Gene	Biological Function
Significantly upregulated		
Abhydrolase domain-containing 10, depalmitoylase	*ABHD10*	A mitochondrial acyl-protein thioesterase modulating mitochondrial antioxidant ability [10].
Actin-related protein 1A	*ACTR1A*	A 42.6 kD subunit of dynactin complex associated with the centrosome and involved in microtubule-based vesicle motility, including ER-to-Golgi transport and the centripetal movement of lysosomes and endosomes. Potential biomarker in pituitary and colon cancers [11,12].
Aminopeptidase N	*ANPEP*	A membrane-bound zinc metalloprotease involved in the metabolism of regulatory peptides. It promotes angiogenesis, tumor growth, and metastasis in melanoma [13,14].
3′(2′), 5′-Bisphosphate nucleotidase 2	*BPNT2*	Member of the inositol monophosphatase family localized to the Golgi apparatus. It catalyzes the hydrolysis of phosphoadenosine phosphate to AMP. No data are available about its role in cancer.
Catenin alpha 1	*CTNNA1*	It connects cadherins located on the plasma membrane to the actin filaments, playing an important role in the cell adhesion process. CTNNA1 germLine variants are associated with hereditary gastric cancer [15].
Cytoplasmic FMR1-interacting protein 1	*CYFIP1*	It regulates cytoskeletal dynamics and protein translation. Involved in tumor metastasis [16].
CXXC motif-containing zinc-binding protein	*CZIB*	Previously referred to as C1orf123, its function remains unknown.
Eukaryotic translation initiation factor 2A	*EIF2A*	It directs the binding of methionyl-tRNAi to 40S ribosomal subunits in a codon-dependent manner. Involved in ER stress in cancer via the (PERK)-eIF2a-ATF4-CHOP signaling axis [17].
Glycogenin 1	*GYG1*	A glycosyltransferase involved in the first steps of glycogen synthesis. A target of miR-194/192 whose expression is downregulated in hepatocellular carcinoma [18].
Karyopherin subunit alpha 4	*KPNA4*	Karyopherins, or importins, are cytoplasmic proteins that recognize NLSs and dock NLS-containing proteins to the nuclear pore complex. Oncosuppressor involved in tumor immune escape [19].
Kynureninase	*KYNU*	It is involved in the biosynthesis of NAD cofactors from tryptophan through the kynurenine pathway. Overexpressed in lung adenocarcinoma, it is associated with immunosuppression and poor survival [20].
Lectin mannose-binding 1 or ER-Golgi intermediate compartment 53 kDa protein (ERGIC-53)	*LMAN1*	Membrane mannose-specific lectin that cycles between the ER, ER-Golgi intermediate compartment, and cis-Golgi, functioning as a cargo receptor for glycoprotein transport. Involved in ER stress and autophagy in human melanoma [21].
MYC-binding protein	*MYCBP*	It binds to the N-terminus of the oncogenic protein C-MYC, enhancing its transcriptional activity. Involved in EMT and progression of triple-negative breast cancer [22].
5′-Nucleotidase ecto	*NT5E*	Plasma membrane protein that catalyzes the conversion of extracellular nucleotides to membrane-permeable nucleosides. Involved in melanoma immune escape via the CD73/adenosine axis [23].
Oxysterol-binding protein	*OSBP*	Lipid transporter involved in lipid counter transport between the Golgi complex and ER membranes. Potential marker for cholangiocarcinoma metastasis [24].
Prolyl 3-hydroxylase 1	*P3H1*	Member of the collagen prolyl hydroxylase family. Localized to the ER, its activity is required for proper collagen synthesis and assembly. Highly expressed by most tumors and associated with overall survival, its knockdown hampers liver cancer cell proliferation, migration, and invasion [25].
Protein O-fucosyltransferase 1	*POFUT1*	Member of the glycosyltransferase O-Fuc family, it adds O-fucose through an O-glycosidic linkage to conserved serine or threonine residues in the epidermal growth factor-like repeats of several cell surface and secreted proteins. Tumor promoter via Notch signaling [26].
PPFIA-binding protein 1	*PPFIBP1*	Member of the LAR protein tyrosine phosphatase-interacting protein (liprin) family. Liprins interact with members of the LAR family of transmembrane protein tyrosine phosphatases. Drives tumor cell migration and invasion via the FAK/Src/JNK pathway [27].
RNA-binding motif protein 12	*RBM12*	It contains several RNA-binding motifs, potential transmembrane domains, and proline-rich regions. It plays a key role in liver cancer immunity [28].
Secreted protein acidic and rich in cysteine	*SPARC*	Cysteine-rich acidic matrix-associated protein. Involved in melanoma metastatic dissemination [29].
Signal recognition particle 72	*SRP72*	72 kDa subunit of the signal recognition particle, a ribonucleoprotein complex that mediates the targeting of secretory proteins to the ER. Involved in epithelial cancers [30].
Transglutaminase 2	*TGM2*	It catalyzes the crosslinking of proteins by epsilon-gamma glutamyl lysine isopeptide bonds. Involved in radioresistance in melanoma [31].
TOR signaling pathway regulator	*TIPRL*	Allosteric regulator of serine/threonine-protein phosphatase 2A. The TIPRL/PP2A axis affects apoptosis and proliferation of cancer cells [32].
X-prolyl aminopeptidase 1	*XPNPEP1*	Cytosolic metalloaminopeptidase that catalyzes the cleavage of the N-terminal amino acid adjacent to a proline residue. Its expression is associated with disease progression and shorter overall survival in multiple myeloma [33].
Zyxin	*ZYX*	A zinc-binding phosphoprotein that concentrates at focal adhesions and along the actin cytoskeleton. It may function as a messenger in the signal transduction pathway that mediates adhesion-stimulated changes in gene expression and may modulate the cytoskeletal organization of actin bundles. Its expression is directly related to melanoma cell spreading and proliferation and inversely related to their differentiation [34].
Significantly downregulated		
Aconitase 2	*ACO2*	It catalyzes the interconversion of citrate to isocitrate via cis-aconitate in the second step of the TCA cycle. Oncosuppressor affecting TCA cycle and mitochondrial oxidative metabolism in cancer cells [35,36].
Serpin family B member 6	*SERPINB6*	A member of the serine proteinase inhibitor superfamily. Its dysregulation is associated with autophagic and apoptotic induction in cancer cells [37].

## Data Availability

The data presented in this study are available in the Supplementary Material.

## Supplementary Materials

The supporting information can be downloaded at: https://www.mdpi.com/article/10.3390/ijms241310555/s1.
