# Supplementary material for: The Pro-Oncogenic Sphingolipid-Metabolizing Enzyme β-Galactosylceramidase Modulates the Proteomic Landscape in BRAF(V600E)-Mutated Human Melanoma Cells
Source: Int J Mol Sci. 2023 Jun 23;24(13):10555. doi: 10.3390/ijms241310555 (PMC10342161; doi:10.3390/ijms241310555)
Supplement: Supplementary file 1 [file ijms-24-10555-s001.zip › Supplementary Figure S1.pdf]

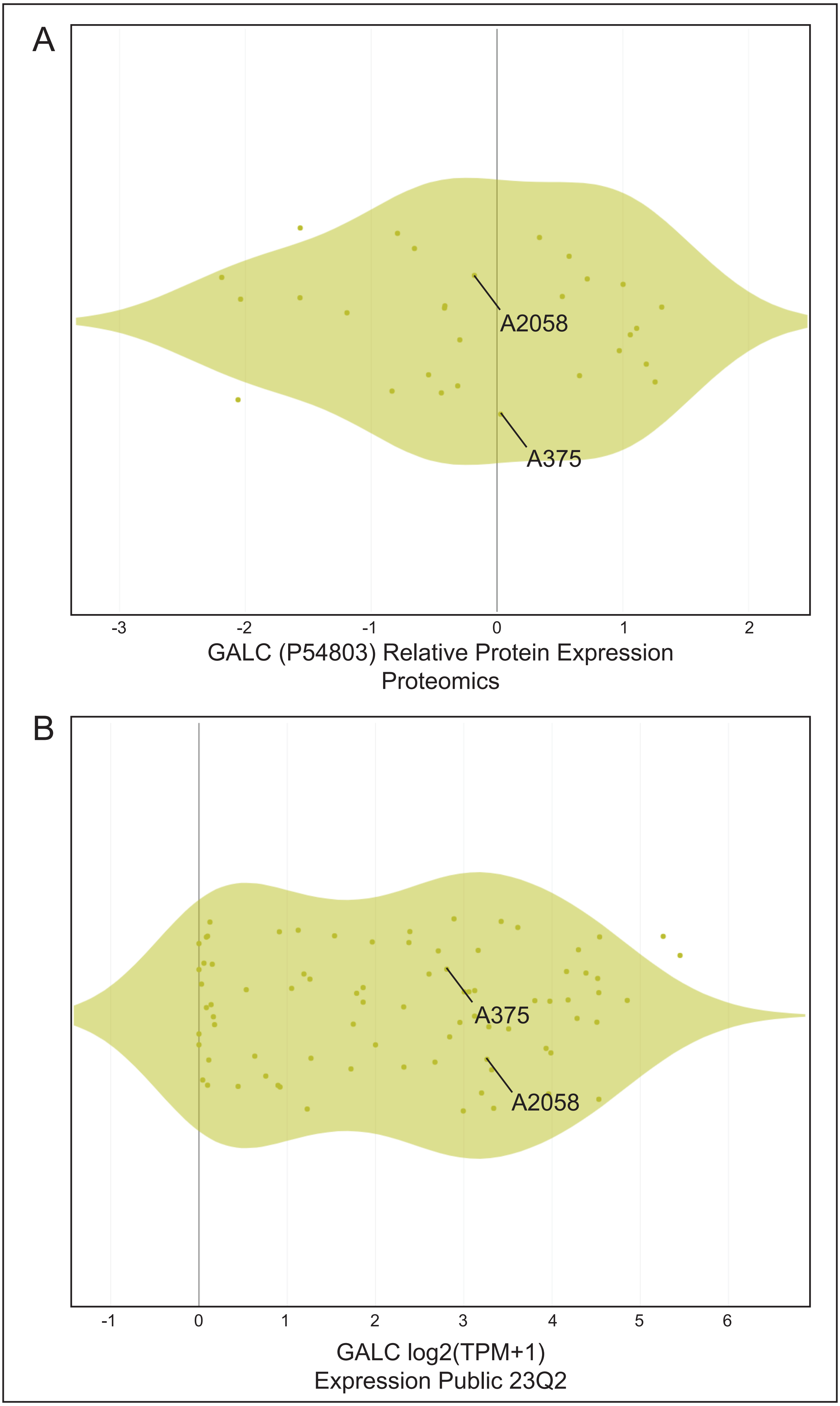

Supplementary Figure S1. GALC expression in human melanoma cel lines. Cancer Dependency Map (DepMap) analysis was performed on 99 human melanoma cell lines (https://depmap.org/). A2058 and A372 cells express intermediate levels of GALC protein (**A**) and *GALC* mRNA (**B**) when compared to the other cell lines.
