# Supplementary material for: The Pro-Oncogenic Sphingolipid-Metabolizing Enzyme β-Galactosylceramidase Modulates the Proteomic Landscape in BRAF(V600E)-Mutated Human Melanoma Cells
Source: Int J Mol Sci. 2023 Jun 23;24(13):10555. doi: 10.3390/ijms241310555 (PMC10342161; doi:10.3390/ijms241310555)
Supplement: Supplementary file 1 [file ijms-24-10555-s001.zip › Supplementary Figure S2.pdf]

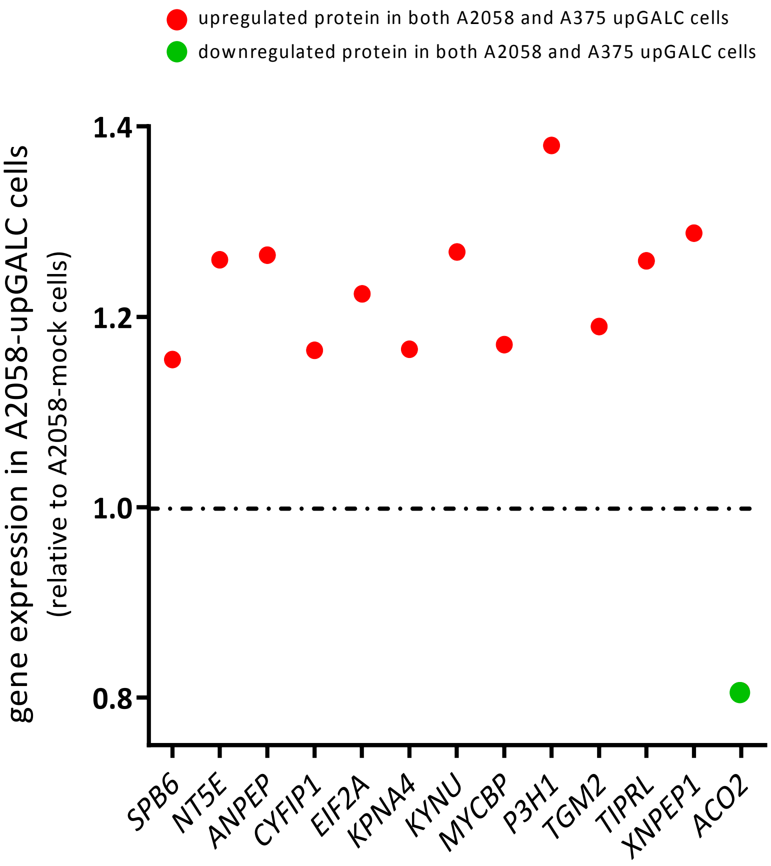

Supplementary Figure S2. RT-qPCR analysis of A2058-upGALC *vs* A2058-mock cells. The expression of genes upregulated (●) or downregulatd (●) by *GALC* overexpression in both A2058 and A375 cells was assessed by RT-qPCR analysis in mock and upGALC A2058 cells. Data are the mean of triplicate values.
