# Supplementary material for: The Pro-Oncogenic Sphingolipid-Metabolizing Enzyme β-Galactosylceramidase Modulates the Proteomic Landscape in BRAF(V600E)-Mutated Human Melanoma Cells
Source: Int J Mol Sci. 2023 Jun 23;24(13):10555. doi: 10.3390/ijms241310555 (PMC10342161; doi:10.3390/ijms241310555)
Supplement: Supplementary file 1 [file ijms-24-10555-s001.zip › Table S4.pdf]

gene	forward	reverse
ACO2	5’-CAATCGTCACCTCCTACAACAGG-3’	5’-GTCTCTGGGTTGAACTTGAGGG-3’
SPB6	5’-CTCCCCGCTCTGGAGTCT-3’	5’-ACAGGACATGCTCATGGGTG-3’
NT5E	5’-TGAACGCAACAATGGCACAAT-3’	5’-GCCTTCTTCAGGGTGGAACC-3’
ANPEP	5’-CCTGATGGACCAGTACAGCG-3’	5’-TCCACTGCTTGAAAAGGCCA-3’
CYFIP1	5’-GACTCACTCGGCCAAGGAAA-3’	5’-CATCCTGGGCTGCGTCC-3’
EIF2A	5’-TCTTTCCGGGACAACATGGC-3’	5’-TCTTCCCAGATTCCCTTGGA-3’
KPNA4	5’-GAAGCTGCATGGGCTTTGAC-3’	5’-AAGTGGCACAGCATTGGACT-3’
KYNU	5’-GGGGGTGCCAGCTAACAATA-3’	5’-TCCGCTTGTCACAAACCACT-3’
MYCBP	5’-ATGGCCCATTACAAAGCCGC-3’	5’-CCAACACCTTGGTCAGCGT-3’
P3H1	5’-CACAGCGAGCGGGACAG-3’	5’-GCTCATCCTTGGGCTTCGAT-3’
TGM2	5’-GTTTGGGGAGATCCAGGGTG-3’	5’-CCACAGCAGTACGTCCCTTC-3’
TIPRL	5’-CTTTTTCCTGCTGTTGCGGT-3’	5’-GGGAAGGTGGAACATGCATCA-3’
XPNPEP1	5’-ACCGCGAGTAAGGGTGAATC-3’	5’-GCATTCTGCCGTCTGTTTCC-3’

Supplementary Table S4. Primers used for RT-qPCR analysis.
